# Microdissection and whole chromosome painting confirm karyotype transformation in cryptic species of the *Lariophagus distinguendus* (Förster, 1841) complex (Hymenoptera: Pteromalidae)

**DOI:** 10.1371/journal.pone.0225257

**Published:** 2019-11-14

**Authors:** Vladimir E. Gokhman, Marcelo de Bello Cioffi, Christian König, Marie Pollmann, Cornelia Gantert, Lars Krogmann, Johannes L. M. Steidle, Nadezda Kosyakova, Thomas Liehr, Ahmed Al-Rikabi

**Affiliations:** 1 Botanical Garden, Moscow State University, Moscow, Russia; 2 Department of Genetics and Evolution, Federal University of São Carlos, São Carlos, São Paulo, Brazil; 3 Institute of Human Genetics, Jena University Hospital, Jena, Germany; 4 Institute for Zoology, University of Hohenheim, Stuttgart, Germany; 5 Department of Entomology, State Museum of Natural History Stuttgart, Stuttgart, Germany; Virginia Polytechnic Institute and State University, UNITED STATES

## Abstract

Karyotypes of two cryptic species of parasitoid Hymenoptera with n = 5 and 6 belonging to the *Lariophagus distinguendus* (Förster, 1841) complex, which includes cosmopolitan parasitoids of coleopteran stored-product pests, were studied using glass-needle based microdissection, reverse and cross-species fluorescence in situ hybridisation (FISH). This experiment strongly indicates that the largest metacentric chromosome in the karyotype with n = 5 originated from a particular fusion between the only acrocentric and a smaller metacentric chromosome of the set with n = 6, therefore confirming our previous hypothesis based on the karyotypic analysis using chromosome morphometrics. This study represents the first successful application of both microdissection and whole chromosome painting for the reconstruction of karyotypic rearrangements in closely related species of parasitoids, as well as in the order Hymenoptera in general.

## Introduction

Parasitoid Hymenoptera are one of the most species-rich, taxonomically complicated and economically important groups of insects [1]. Specifically, its world fauna contains more than 80 thousand described species [2]. However, karyotypes of only about 500 members of this group have been examined up to now [3]. Moreover, transformations of parasitoid karyotypes are poorly studied, mainly due to the lack of corresponding research by appropriate methods [4]. A few recent studies involving chromosome morphometrics (e.g. [5]) identified certain putative karyotypic rearrangements distinguishing closely related parasitoid species. Nevertheless, these results were rarely confirmed by any molecular technique [5].

Glass-needle based chromosome microdissection (midi) is a powerful tool for characterization of karyotypic transformations, both intra- and interspecific ones [6–8]. For example, a particular microdissected chromosome applied as “whole chromosome painting” (WCP) probe in fluorescence in situ hybridisation (FISH) derived from species A is able to identify homologous segments of another chromosome in species B, thus e.g. providing reliable evidence for a particular chromosomal fusion or fission [9–10]. Midi-derived probes applied for reverse and cross-species FISH (ZOO-FISH) are now widely used to analyse karyotype evolution in many groups of insects and other arthropods (see e.g. [11]). Moreover, the WCP technique has proven to be an excellent tool for identifying chromosomal rearrangements involved in the karyotype evolution of certain insect taxa [12–14].

However, chromosome sets of quite a few members of the order Hymenoptera were studied using the above-mentioned techniques. Specifically, microdissection was applied to a few species belonging to the family Apidae, i.e. bees [15–17] (reviewed in [18]). Nevertheless, the karyotype of the only parasitoid species, *Nasonia vitripennis* (Walker, 1836) (Chalcidoidea: Pteromalidae), was analysed using the aforementioned approach, i.e. midi and reverse FISH [19–20]. Although it was possible to identify and characterize all chromosomes of this well-known species with n = 5, no further conclusions regarding its karyotype evolution were drawn.

*Lariophagus distinguendus* (Förster, 1841), which also belongs to the family Pteromalidae, is an apparently well-studied cosmopolitan parasitoid of certain beetle species (Coleoptera) which feed on many stored products [21–22]. However, our recent research revealed the presence of at least two cryptic species with alternative host preferences and strongly different DNA sequences [23–25]. Although these species can hybridise under certain conditions, they also have different karyotypes with n = 5 and 6 [24–25]. Moreover, morphometrics of routinely stained chromosomes, together with a recent cladistic analysis of different populations of *L*. *distinguendus*, suggests that the latter karyotype represents an ancestral character state, and, consequently, the chromosome set with n = 5 has undergone a particular chromosomal fusion [25]. Accordingly, the largest metacentric chromosome (M) has apparently resulted from this fusion, most likely involving the only acrocentric (A) and a smaller metacentric in the chromosome set of the cryptic species with n = 6. However, this scenario was never analysed by means of molecular genetics. The aim of the present work is therefore to test the hypothesis of the putative rearrangements in the *L*. *distinguendus* species complex previously suggested by chromosome morphometrics using microdissection and whole chromosome painting.

## Materials and methods

### Origin of parasitoids

**Fig 1:**
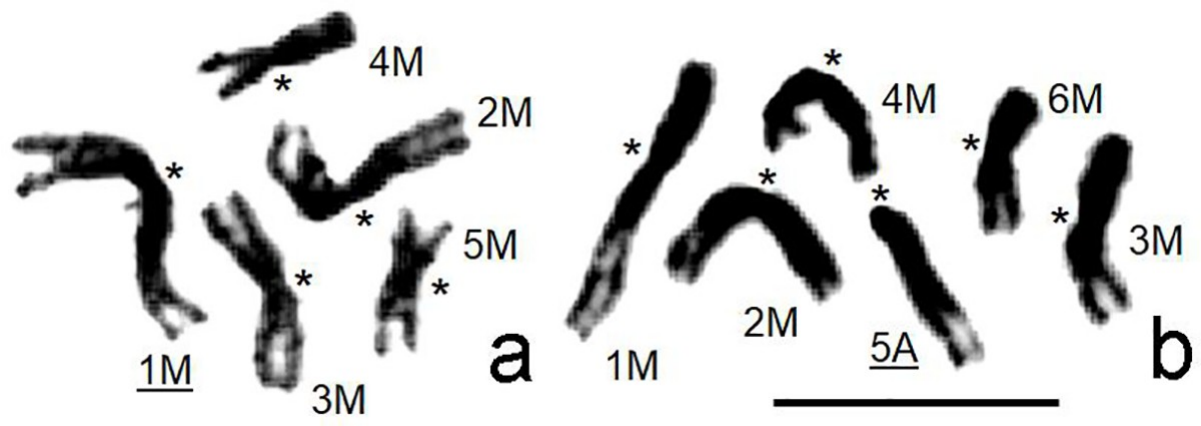
Haploid metaphase plates from dividing neuroblasts of male cerebral ganglia of *Lariophagus distinguendus*. (n = 5) (a); (n = 6) (b). Giemsa staining. Chromosomes are labelled according to their lengths and centromere positions (indicated by asterisks), labels of microdissected chromosomes are underlined. Bar = 10 μm.

Two strains of *L*. *distinguendus*, PFO-D and RAV-D, which were respectively collected in Pforzheim and Ravensburg (Baden-Württemberg, Germany) and kept as lab stocks at the Institute for Zoology, University of Hohenheim, Stuttgart, Germany [23–25] were used in the present study. Identification of all parasitoids was performed by Lars Krogmann on the basis of the morphological description of *L*. *distinguendus* (see e.g. [26]). Our previous work [25] demonstrated that the PFO-D and RAV-D strains have n = 5 and 6 chromosomes respectively (Fig 1), and therefore we refer to them as “*L*. *distinguendus* (n = 5)” and “*L*. *distinguendus* (n = 6)” in the present paper.

### Techniques for obtaining chromosomal preparations and probes

Chromosomal preparations were obtained from cerebral ganglia of male and female parasitoid prepupae using the protocol developed by [27] with certain modifications which follow [25]. Specifically, ganglia were extracted from insects dissected in 0.5% hypotonic sodium citrate solution containing 0.005% colchicine. The extracted ganglia were then transferred to a fresh portion of hypotonic solution and incubated for 30 min at room temperature (RT). The material was transferred onto a pre-cleaned microscope slide (or a 24 × 60 mm coverslip) using a Pasteur pipette and then gently flushed with Fixative I (glacial acetic acid: absolute ethanol: distilled water 3:3:4). The tissues were disrupted using dissecting needles in an additional drop of Fixative I. Another drop of Fixative II (glacial acetic acid: absolute ethanol 1:1) was applied to the centre of the area, and the more aqueous phase was blotted off the edges of the slide. The slides were then dried for approximately half an hour and stored at RT. Certain preparations of both strains were preliminarily stained in a freshly prepared 3% Giemsa solution (Merck, Kenilworth, New Jersey, USA) in 0.05M Sorensen’s phosphate buffer (Na_2_HPO_4_ + KH_2_PO_4_, pH 6.8).

Twelve copies of the largest M chromosome of *L*. *distinguendus* (n = 5) and 14 copies of the only A chromosome of *L*. *distinguendus* (n = 6) were obtained by glass-needle based chromosome microdissection [28]. To perform microdissection, target chromosomes were isolated from metaphase plates using a micromanipulator controlled by an inverted microscope Zeiss Axiovert 10 (Carl Zeiss AG, Oberkochen, Germany).

The probes obtained from *L*. *distinguendus* (n = 5 and 6), designated as LD5-1 and LD6-A respectively, were then amplified according to [28]. To amplify the probes, primary DOP-PCR (degenerate oligonucleotide-primed polymerase chain reaction) was used. Specifically, a collection drop containing the microdissected chromosomes was transferred into a tube with 5 μL PCR master mix containing 0.6 μL 5× T7 DNA Polymerase Reaction Buffer (Thermo Fisher Scientific, Waltham, Massachusetts, USA), 0.016 mM dNTP, 4.8 μM DOP primer, and 3.4 μL PCR water. After adding the PCR master mix, the low-temperature cycle (LTC) of PCR was performed (5 min at 92°C, 2 min 20 s at 25°C, 2 min at 34°C, and 1 min at 90°C). During the second step of LTC, 0.2 μL T7 DNA Polymerase mix [0.025 μL T7 DNA Polymerase 10 U μL^-1^ and 0.175 μL T7 DNA Polymerase Dilution Buffer (Thermo Fisher Scientific)] was added, and the whole cycle except for the first step was repeated another seven times. After that, 45 μL of the PCR mix for high-temperature cycles (HTC) containing 28.0 μL water, 5.0 μL 10× AmpliTaq Gold DNA Polymerase Buffer, 0.222 mM dNTP, 2.778 mM MgCl_2_, 0.622 μM DOP primer, 0.6 μL 5 U μL^-1^ AmpliTaq Gold DNA Polymerase was added and HTC-PCR was performed. The latter procedure (1 min at 92°C, 2 min at 56°C, and 2 min at 72°C) was repeated 33 times, followed by another 2 min at 72°C and stopped by keeping the mixture at 4°C.

The probes LD5-1 and LD6-A were then respectively labelled with Spectrum Orange-dUTP and Spectrum Green-dUTP (Vysis, Downers Grove, Illinois, USA) in a secondary DOP-PCR using the procedure described in [28]. Specifically, 1.0 μL each primary DOP-PCR product was added to 20.0 μL of the following master mix: 10.9 μL water, 1.0 μM DOP primer, 0.2 mM d(A,C,G)TP, 0.1 mM dTTP, 2.5 mM MgCl_2_, 0.1 mM labelled dUTP, 0.1 μL 5 U μL^-1^ AmpliTaq Gold DNA Polymerase. The PCR procedure included 3 min at 95°C, followed by 20 amplification cycles (1 min at 94°C, 1 min at 56°C, 2 min at 72°C), another 5 min at 72°C and stopped by keeping the mixture at 4°C. The PCR product was then precipitated in ethanol, centrifugated at 13,000 rpm for 20 min, and resuspended in 20 μL hybridisation buffer (see below).

### FISH experiments

Chromosomal preparations of *L*. *distinguendus* (n = 5 and 6) were used for the bidirectional reverse FISH and ZOO-FISH experiments, that were carried out following [29]. The preparations were first dehydrated in ethanol (70, 90 and 100% for 2 min each), air-dried at 60°C for 1 h, and treated with 100 μL of RNase A (10 μg mL^-1^) under 24 × 60 mm coverslips for 1 h at 37°C in a moisture chamber. After that, the slides were treated with 1× phosphate buffered saline (PBS, Biochrom, Cambridge, UK) for 5 min on a shaker at RT, and this procedure was also repeated after each of the two following steps, i.e. treatment with 0.005% pepsin solution under a coverslip for 10 min at 37°C, and post-fixation with 1% paraformaldehyde solution (50 mL 2% paraformaldehyde plus 45 mL 1× PBS and 5 mL 1 M MgCl_2_) for 10 min at RT. The preparations were then dehydrated in ethanol (see above) and air-dried.

Prior to in situ hybridisation, the preparations were incubated in a formamide solution (2× saline-sodium citrate (SSC) buffer (Merck), 50% deionized formamide, pH 7.0) for 3 min at 75°C, then dehydrated in cold ethanol (-20°C; see above) and air-dried. The hybridisation mix containing 20 μL of the hybridisation buffer (10 mg formamide and 4 mg dextran sodium sulphate in 2× SSC and 0.1 mM phosphate buffer) and 100 ng of the labelled probe was then denatured in a thermocycler for 10 min at 85°C, applied to the preparation, covered with a coverslip and incubated for 14 h at 37°C in a dark moisture chamber. After removing the coverslip, the slide was rinsed in 1× SSC at 65°C. The slide was then rinsed in a washing buffer (4× SSC containing 0.05% Tween 20) for 5 min at RT on a shaker, dehydrated in ethanol (see above), air-dried and mounted in Vectashield Mounting Medium with DAPI (Vector Laboratories, Burlingame, CA, USA).

### Isolation of C_0_t-1 DNA

Since initial experiments demonstrated substantial cross-hybridisation of both probes LD5-1 and LD6-A to non-target chromosomes due to the presence of high-copy repeat sequences, C_0_t-1 DNA was applied to block these sequences. To prepare C_0_t-1 DNA, genomic DNA was first extracted from ten unsexed pupae of each species according to [30] with a few modifications. Specifically, pupae were crushed in a small tube with a bead, and then homogenised in 1 mL of ice-cold buffer A (0.35 M sucrose, 0.05 M Tris–HCl, pH 7.5, 0.066 M EDTA, 0.003 M CaCl_2_, 0.025 M KCl). After slowly adding 10% solution of Triton X-100 in buffer A to the final concentration of 1%, the mix was centrifugated three times at 800× g and 4°C for 5 min, with the supernatant discarded and the pellet resuspended in 1 mL of ice-cold buffer A every time. After final resuspension, 4 mL of buffer B (0.05 M Tris–HCl, pH 7.5, 0.066 M EDTA, 0.1 M NaCl) and RNase A (to the final concentration 10 mg mL^-1^) at RT was added. After this step, equal amounts (2.5 mL) of 0.4% sodium dodecyl sulphate (SDS) and 0.1 mg mL^-1^ of proteinase K (Thermo Fisher Scientific) in buffer B were added, and the mixture was stirred. It was then incubated for 1 h at 60°C and vortexed every 20 min.

In turn, C_0_t-1 DNA was isolated following the procedure described in [31]. Specifically, the genomic DNA was diluted to a concentration between 100 and 500 ng μL^-1^ using 5 M NaCl and double-distilled H_2_O to a final concentration of 0.3 M NaCl. Then the DNA was sheared by autoclaving it several times on a liquid cycle (5 min each), until the necessary fragment size of 100 to 1,000 bp (controlled by electrophoresis) was reached. After denaturing in a water bath at 95°C for 10 min and cooling in ice water for 10 s, DNA was reannealed by placing it again in a water bath at 65°C. To digest the remaining single-stranded DNA, calculated amounts of l0× S1 nuclease buffer (0.5 M NaOAc, pH 4.9, 45 mM ZnSO_4_) (Merck) and S1 nuclease (Thermo Fisher Scientific) were then added to achieve the concentration of 1 U S1 nuclease μg^-1^ DNA, and the mix was immediately put in a water bath at 37°C for 8 min. The nuclease digestion was then stopped by DNA extraction with phenol equilibrated with Tris-EDTA (TE) buffer, and the supernatant was further extracted with an equal volume of phenol-chloroform followed by an equal volume of chloroform (4% of isoamyl alcohol were added to chloroform in both cases). C_0_t-1 DNA was then precipitated overnight using 2.5 volumes of 100% ethanol, dried, resuspended in TE buffer and stored at -20°C until needed. Different amounts of C_0_t-1 DNA, i.e. 20 and 70 μg, were added to the hybridisation mix, with the latter amount appeared to yield the best results.

### Image acquisition and processing

Chromosomes were visualized using an epifluorescence microscope Zeiss Axioplan (Carl Zeiss AG) fitted with a digital CCD camera Olympus DP70 (Olympus Corporation, Tokyo, Japan). Additional images of Giemsa-stained chromosomes were obtained using an optic microscope Zeiss Axioskop 40 FL fitted with a digital CCD camera Zeiss Axiocam MRc (Carl Zeiss AG). Chromosomes were classified as M or A according to their arm ratios [32]. Images were obtained, enhanced and arranged using Isis (MetaSystems GmbH, Altlussheim, Germany), Zeiss AxioVision 3.1 (Carl Zeiss AG) and Adobe Photoshop 7.0 and 8.0 (Adobe Inc., San Jose, California, USA) software.

## Results and discussion

**Fig 2:**
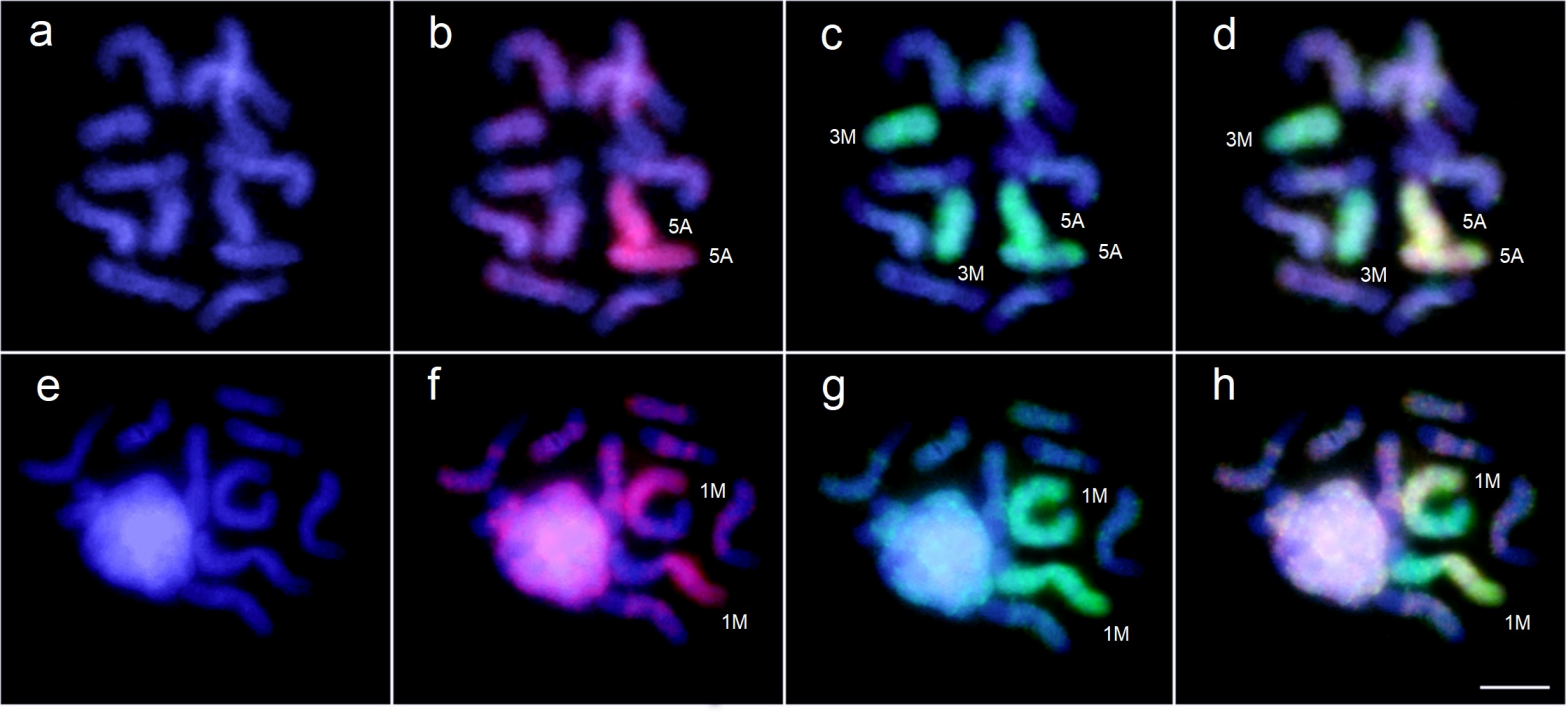
Fluorescence in situ hybridisation of LD6-A and LD5-1 probes with metaphase chromosomes from dividing diploid neuroblasts of female cerebral ganglia of *Lariophagus distinguendus*. (n = 6) (a–d) and (n = 5) (e–h). LD6-A and LD5-1 probes display red and green signals respectively. Chromosomes carrying hybridisation signals are labelled according to their lengths and centromere positions (see Fig 1). (a, e) DAPI staining; (b, f) hybridisation with LD6-A; (c, g) hybridisation with LD5-1; (d, h) merged images. Bar = 10 μm.

**Fig 3:**
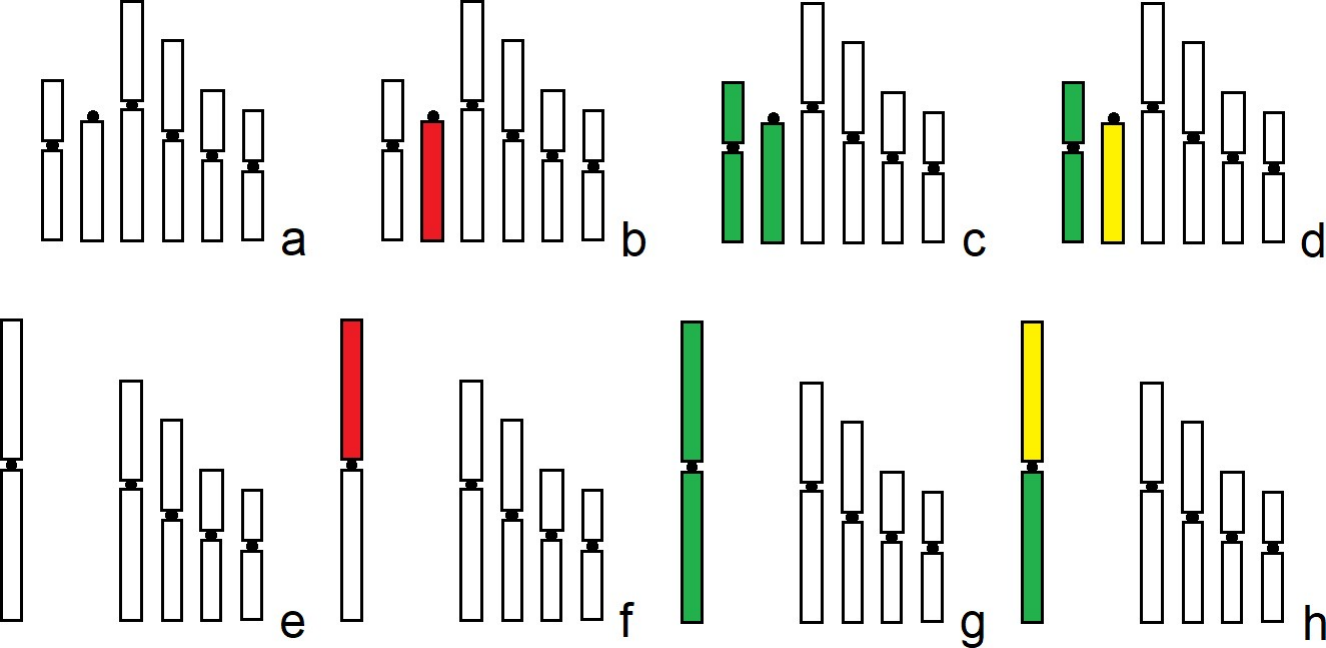
Schematic representation of chromosome sets of *Lariophagus distinguendus* presented on Fig 2. Only haploid chromosome sets are shown. Superposition of signals from both probes is indicated in yellow. Relative lengths and centromere indices of chromosomes are based on data listed in [25].

The results of the FISH experiments are shown on Fig 2 (see also Fig 3 for schematic representations of the corresponding micrographs). The experiments with the chromosome set of *L*. *distinguendus* (n = 6) demonstrated that LD6-A probe hybridised with the only A pair (Fig 2B), while LD5-1 probe also marked these chromosomes as well as another relatively small chromosome pair (Fig 2C). In turn, an analogous study of the karyotype of *L*. *distinguendus* (n = 5) showed that LD5-1 probe strongly painted the largest M pair (Fig 2G), whereas LD6-A probe hybridised only with the shorter arms of the same pair of chromosomes (Fig 2F). In addition, faint hybridisation signals from both probes were observed in the heterochromatic regions of several other chromosome pairs (see e.g. Fig 2F–2H), which can be explained by a higher amount of apparently species-specific subset of heterochromatin in these parasitoids.

The results obtained clearly show that the largest M pair in the chromosome set of *L*. *distinguendus* (n = 5) resulted from a chromosomal fusion of the unique pair of acrocentrics and a smaller M chromosome pair in the karyotype of *L*. *distinguendus* (n = 6), thus confirming our previous assumptions on the type and direction of karyotype transformation in this complex respectively made on the basis of chromosome morphometrics and cladistic analysis of different populations [25]. It therefore demonstrates the significance of the morphometric study as a cost-effective approach for analysing karyotypes of parasitoid Hymenoptera, especially those with lower chromosome numbers [5]. To our knowledge, this work represents the first successful use of midi and whole chromosome painting for studying karyotype rearrangements in closely related parasitoid species. Moreover, this also applies to the order Hymenoptera in general, since the only karyotypic study of a non-parasitoid member of this group with the help of WCPs [16] used reverse FISH to distinguish between different types of B chromosomes in a particular member of the family Apidae. It is also noteworthy that whole chromosome painting, in fact, apparently represents the most reliable means of recognizing homologous chromosomes in closely related hymenopteran species. Indeed, G-banding can identify all chromosomes within the given set in certain chalcids, e.g. *Nasonia vitripennis* [19–20] or a few members of the genus *Encarsia* Förster, 1878 (Aphelinidae) [33–34], although the possibility to recognize homologous chromosomes within karyotypes of distantly related species using this technique is unclear (see [4] for review). This situation, which substantially differs from that characteristic of vertebrates, could be explained by the different structure of chromosomes in the latter group, as opposed to insects [35]. We therefore consider the present research a model case study aimed for reconstructing karyotype evolution within various groups of the order Hymenoptera, as it is already done for many well-studied animal taxa, e.g. mammals [9]. We also believe that the technique described in the present work can be especially useful for analysing chromosomal rearrangements between hymenopteran karyotypes with higher chromosome numbers, because morphometric study of many similar-sized chromosomes appears less effective for that purpose.
